# Supplementary material for: A comprehensive Bioconductor ecosystem for the design of CRISPR guide RNAs across nucleases and technologies
Source: Nat Commun. 2022 Nov 2;13:6568. doi: 10.1038/s41467-022-34320-7 (PMC9630310; doi:10.1038/s41467-022-34320-7)
Supplement: Supplementary file 1 — Supplementary Information [file 41467_2022_34320_MOESM1_ESM.pdf]

A comprehensive Bioconductor ecosystem for the design of CRISPR guide RNAs across nucleases and technologies

Supplementary information

October 20, 2022

Supplementary Tables

Target	Spacer specification			Number of mismatches											
Gene	Coordinate	Spacer	PAM	0			1			2			3		
				AC	BO	BW	AC	BO	BW	AC	BO	BW	AC	BO	BW
CFTR	chr7:117559595	ATTAAAGAAAATATCATCTT	TGG	1	1	1	7	7	7	145	145	145	2,314	2,314	2,314
	chr7:117559605	TCTGTATCTATATTCATCAT	AGG	1	1	1	7	7	7	125	125	125	1,704	1,704	1,704
HBB	chr11:5227002	CATGGTGCATCTGACTCCTG	AGG	2	2	2	0	0	0	14	14	14	210	210	210
	chr11:5227004	GTAACGGCAGACTTCTCCTC	AGG	1	1	1	0	0	0	7	7	7	83	83	83
HEXA	chr15:72346571	TGTAGAAATCCTTCCAGTCA	GGG	1	1	1	0	0	0	25	25	25	298	298	298
	chr15:72346578	ATCCTTCCAGTCAGGGCCAT	AGG	1	1	1	0	0	0	6	6	6	203	203	203
PRNP	chr20:4699588	AGCAGCTGGGGCAGTGGTGG	GGG	1	1	1	2	2	2	96	96	96	909	909	909
	chr20:4699589	GCAGCTGGGGCAGTGGTGGG	GGG	1	1	1	12	12	12	100	100	100	1,052	1,052	1,052
	chr20:4699595	GGGGCAGTGGTGGGGGGCCT	TGG	1	1	1	2	2	2	56	56	56	860	860	860
	chr20:4699598	GCAGTGGTGGGGGGCCTTGG	CGG	1	1	1	0	0	0	32	32	32	421	421	421

Supplementary Table 1. Table of on- and off-target alignments in the GRCh38.p13 for the 10 spacer sequences reported in **(author?)** [1] using a PAM-agnostic approach. Number of mismatches between 0 and 3 were considered for 3 different aligners: Aho-Corasick exact string matching as implemented in *Biostrings* (AC), *Bowtie* aligner via the *crisprBowtie* package (BO), and *BWA* aligner via the *crisprBwa* package (BW). All 3 alignment methods agree.

Supplementary Figures

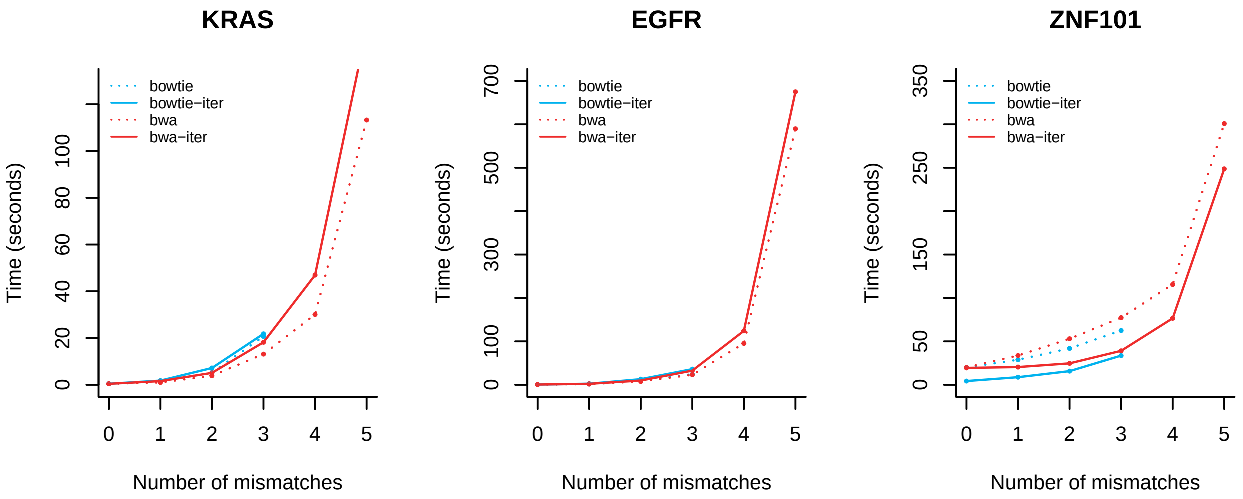

Supplementary Figure 1. Comparison of computing times for off-target alignment methods implemented in *crisprDesign*. We compare computing time for the four different off-target methods available via the *addSpacerAligmments* function in *crisprDesign*: *Bowtie*, via the *crisprBowtie* package (`Bowtie`), *BWA*, via the *crisprBwa* package (`bwa`), and an iterative version of both algorithms to diminish the impact of highly non-specific gRNAs on computing time (`bowtie-iter` and `bwa-iter`). Source data are provided as a Source Data file.

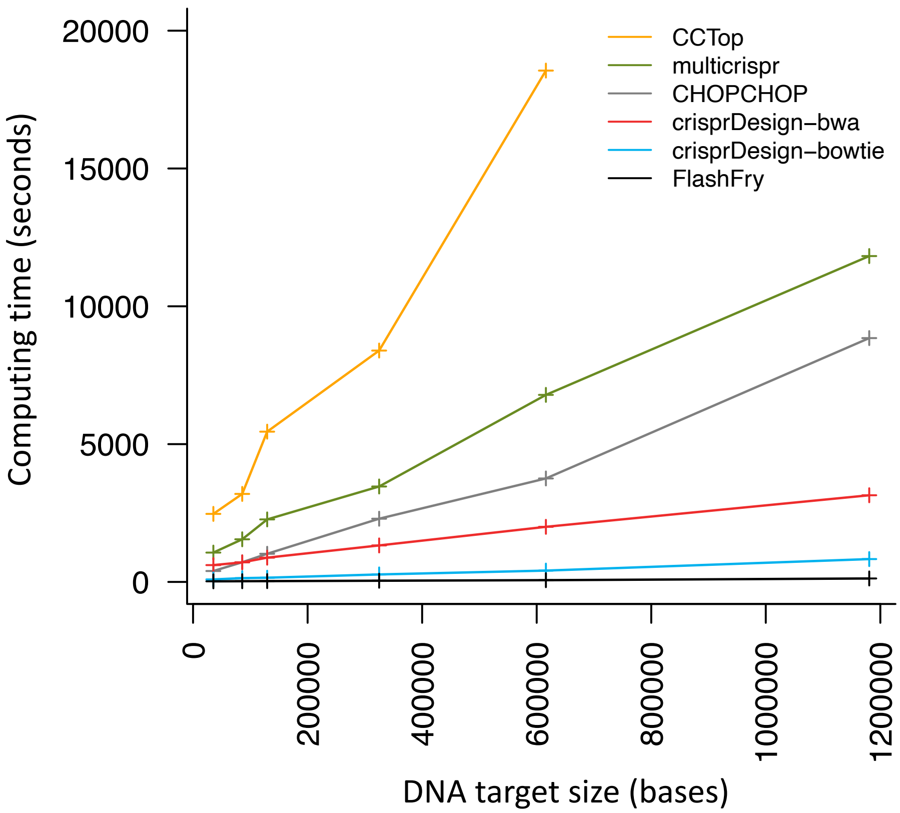

Supplementary Figure 2. Comparison of computing times for subsets of human protein-coding exons. We compared computing times across tools to design gRNAs and perform a genome-wide off-target search in the human genome. Six random subsets of protein-coding exons located on chr1 were used to perform the comparison. The sizes of the subsets were 100, 200, 400, 800, 1600 and 3200 exons. The x-axis shows the total size in nucleotides of the DNA target space formed by each subset, and the y-axis shows computing times in seconds. Details about the alignment parameters for each method can be found in the Methods section. Source data are provided as a Source Data file.

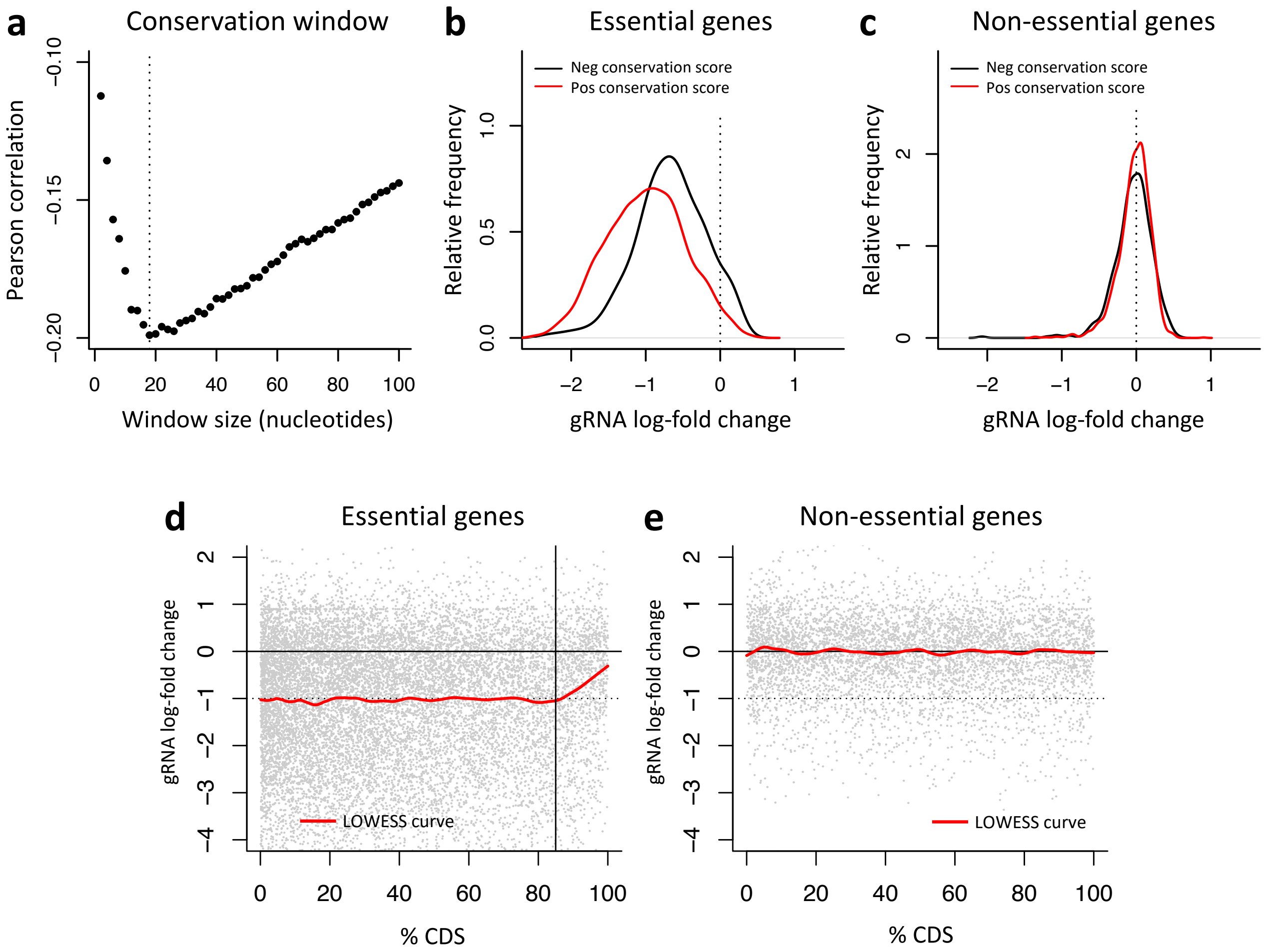

Supplementary Figure 3. Influence of evolutionary conservation and gene target position on gRNA activity. a-c We annotated each gRNA present in Project Achilles with a conservation score using the function *addConservationScores* implemented in *crisprDesign* (see Methods). The gRNA conservation score is taken as the average DNA conservation score across nucleotides in a user-specified window around the gRNA cut site. In **a**, we show the correlation between observed gRNA activity and the conservation scores for different window sizes for essential genes. The data suggest an optimal window of 18 nucleotides around the cut site. **b** Distributions of the observed gRNA log-fold changes (LFCs) based on whether or not gRNAs are targeting regions of high conservation (positive gRNA conservation score) or regions of low conservation (negative gRNA conservation score), for gRNAs targeting essential genes. **c** Same as **b**, but for gRNAs targeting non-essential genes. **d** Relationship between gRNA activity and gRNA position within the target coding sequence (CDS) for gRNAs targeting essential genes in the Hart2015 dataset. The Ensembl canonical transcript was used as the target CDS for each gene. The red curve represents a LOWESS trend. gRNAs located beyond the first 85% of the CDS (to the right of the the vertical line) show a progressive decline in activity. **e** Same as **d**, but for gRNAs targeting non-essential genes. Source data are provided as a Source Data file.

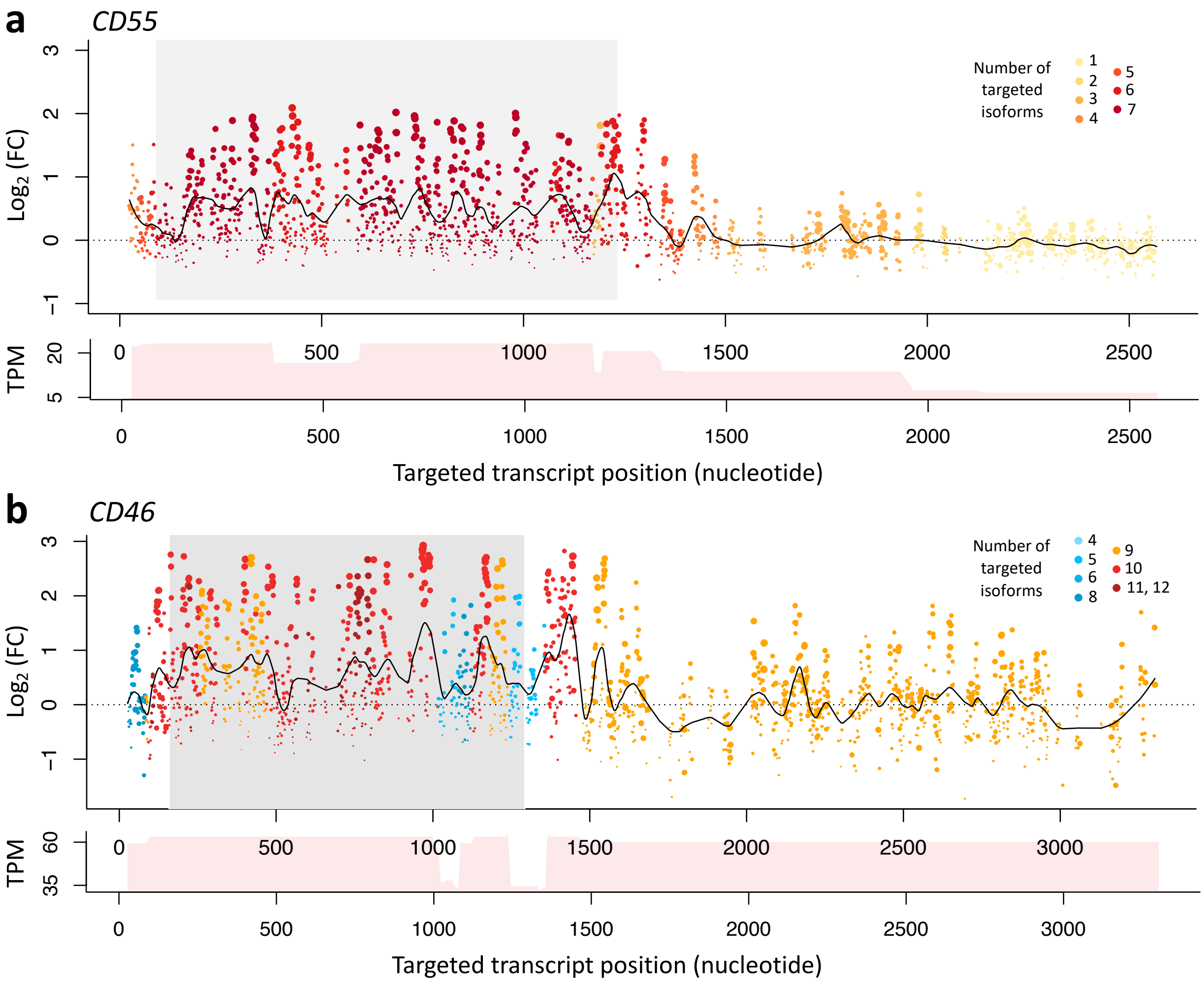

Supplementary Figure 4. CasRx tiling screens of *CD55* and *CD46*. Pooled FACS tiling screening data of genes *CD55* (**a**) and *CD46* (**b**) performed in HEK 294 cells using CasRx (*Rfx*Cas13d). Processed and normalized $\log_2$ fold changes were obtained from **(author?)** [2]. Both screens are represented using the canonical Ensembl isoforms. We remapped and reannotated all gRNA sequences using *crisprDesign*; isoform annotation, on-target activity score using CasRx-RF as implemented in *crisprScore*, and off-target alignments were added to each gRNA. The color of the dots indicates the number of isoforms targeted by each gRNA. The size of the dots is proportional to the on-target activity score. The coding sequence (CDS) is highlighted in grey. LOESS regression curves are shown as solid lines. For both genes, transcript per million (TPM) counts in HEK 293 cells summed across all isoforms overlapping a given nucleotide position are shown below the log-fold change panels. Source data are provided as a Source Data file.

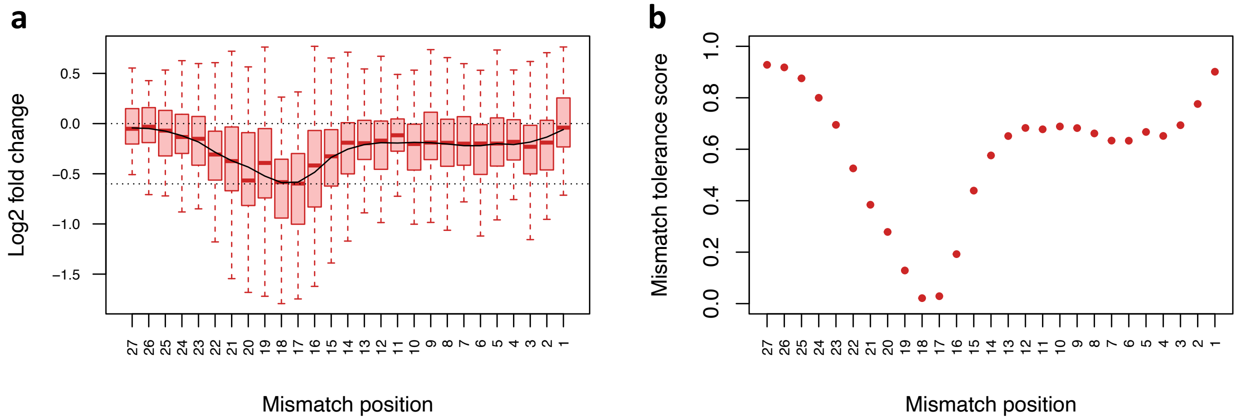

Supplementary Figure 5. Probability weights used for off-target scoring of CasRx gRNAs a Boxplots of the differences in log2 fold change (ΔLFC) between single-mismatch (SM) gRNAs and their corresponding perfect-match (PM) gRNAs in the GFP tiling screen. The boxes represent the $25 - 75\%$ interquartile ranges (IQR), and the central lines represent the median values. The whiskers extend 1.5 times the IQR from the median value. Each boxplot contains $n = 78$ data points. X-axis represents the mismatch position within the spacer sequence, with 1 being the position next to the direct repeat. The smooth curve was obtained using LOESS regression. The dotted line represents the average log-fold change of all PM gRNAs after multiplying by -1. **b** CasRx mismatch tolerance probabilities estimated from (a) and used in the CFD scoring method in *crisprScore*. Source data are provided as a Source Data file.

References

[1] Bhagwat, A. M. *et al.* multicrispr: grna design for prime editing and parallel targeting of thousands of targets. *Life science alliance* **3** (2020).

[2] Wessels, H.-H. *et al.* Massively parallel cas13 screens reveal principles for guide rna design. *Nature biotechnology* **38**, 722–727 (2020).
