# Supplementary material for: A comprehensive Bioconductor ecosystem for the design of CRISPR guide RNAs across nucleases and technologies
Source: Nat Commun. 2022 Nov 2;13:6568. doi: 10.1038/s41467-022-34320-7 (PMC9630310; doi:10.1038/s41467-022-34320-7)
Supplement: Supplementary file 3 — Description of Additional Supplementary Files [file 41467_2022_34320_MOESM3_ESM.pdf]

Title: Supplementary Software file

Description: R package vignettes and tutorials for the crisprVerse ecosystem.
