# Supplementary material for: A comprehensive Bioconductor ecosystem for the design of CRISPR guide RNAs across nucleases and technologies
Source: Nat Commun. 2022 Nov 2;13:6568. doi: 10.1038/s41467-022-34320-7 (PMC9630310; doi:10.1038/s41467-022-34320-7)
Supplement: Supplementary file 5 — Supplementary Software [file 41467_2022_34320_MOESM5_ESM.zip › SupplementarySoftware/SoftwareVignette8_crisprVerse.pdf]

crisprVerse: ecosystem of R packages for CRISPR gRNA design

Jean-Philippe Fortin, Luke Hoberecht

2022-10-17

1 Installation and getting started

The crisprVerse is a collection of packages for CRISPR guide RNA (gRNA) design that can easily be installed with the `crisprVerse` package. This provides a convenient way of downloading and installing all crisprVerse packages with a single R command.

The package can be installed from the Bioconductor devel branch using the following commands in an R session:

```
if (!requireNamespace("BiocManager", quietly = TRUE))
    install.packages("BiocManager")

BiocManager::install(version="devel")
BiocManager::install("crisprVerse")
```

The core crisprVerse includes the packages that are commonly used for gRNA design, and are attached when you attach the `crisprVerse` package:

```
library(crisprVerse)
```

```
## Warning: multiple methods tables found for 'aperm'
```

```
## Warning: replacing previous import 'BiocGenerics::aperm' by
## 'DelayedArray::aperm' when loading 'SummarizedExperiment'
```

You can check that all crisprVerse packages are up-to-date with the function `crisprVerse_update()`.

2 Components

The following packages are installed and loaded with the `crisprVerse` package:

- *crisprBase* to specify and manipulate CRISPR nucleases.
- *crisprBowtie* to perform gRNA spacer sequence alignment with Bowtie.
- *crisprScore* to annotate gRNAs with on-target and off-target scores.
- *crisprDesign* to design and manipulate gRNAs with `GuideSet` objects.

3 Reproducibility

```
sessionInfo()
```

```
## R version 4.2.1 (2022-06-23)
## Platform: x86_64-apple-darwin17.0 (64-bit)
## Running under: macOS Catalina 10.15.7
##
```

```
## Matrix products: default
## BLAS:   /Library/Frameworks/R.framework/Versions/4.2/Resources/lib/libRblas.0.dylib
## LAPACK: /Library/Frameworks/R.framework/Versions/4.2/Resources/lib/libRlapack.dylib
##
## locale:
## [1] en_US.UTF-8/en_US.UTF-8/en_US.UTF-8/C/en_US.UTF-8/en_US.UTF-8
##
## attached base packages:
## [1] stats     graphics  grDevices utils     datasets  methods   base
##
## other attached packages:
##  [1] crisprViz_0.99.22     crisprDesign_0.99.176 crisprScore_1.1.15
##  [4] crisprScoreData_1.1.3 ExperimentHub_2.5.0   AnnotationHub_3.5.1
##  [7] BiocFileCache_2.5.0   dbplyr_2.2.1          BiocGenerics_0.43.4
## [10] crisprBowtie_1.1.1    crisprBase_1.1.8      crisprVerse_0.99.9
##
## loaded via a namespace (and not attached):
##   [1] backports_1.4.1              Hmisc_4.7-1
##   [3] lazyeval_0.2.2               splines_4.2.1
##   [5] BiocParallel_1.31.12         GenomeInfoDb_1.33.7
##   [7] ggplot2_3.3.6                digest_0.6.29
##   [9] ensembldb_2.21.4             htmltools_0.5.3
##  [11] fansi_1.0.3                  checkmate_2.1.0
##  [13] magrittr_2.0.3               memoise_2.0.1
##  [15] BSgenome_1.65.2              cluster_2.1.4
##  [17] tzdb_0.3.0                   Biostrings_2.65.3
##  [19] readr_2.1.2                  matrixStats_0.62.0
##  [21] prettyunits_1.1.1            jpeg_0.1-9
##  [23] colorspace_2.0-3             blob_1.2.3
##  [25] rappdirs_0.3.3               xfun_0.32
##  [27] dplyr_1.0.10                 crayon_1.5.1
##  [29] RCurl_1.98-1.8               jsonlite_1.8.0
##  [31] survival_3.4-0               VariantAnnotation_1.43.3
##  [33] glue_1.6.2                   gtable_0.3.1
##  [35] zlibbioc_1.43.0              XVector_0.37.1
##  [37] DelayedArray_0.23.1          scales_1.2.1
##  [39] DBI_1.1.3                    Rcpp_1.0.9
##  [41] htmlTable_2.4.1              xtable_1.8-4
##  [43] progress_1.2.2               reticulate_1.26
##  [45] foreign_0.8-82               bit_4.0.4
##  [47] Formula_1.2-4                stats4_4.2.1
##  [49] htmlwidgets_1.5.4            httr_1.4.4
##  [51] dir.expiry_1.5.1             RColorBrewer_1.1-3
##  [53] ellipsis_0.3.2               pkgconfig_2.0.3
##  [55] XML_3.99-0.10                Gviz_1.41.1
##  [57] nnet_7.3-17                  deldir_1.0-6
##  [59] utf8_1.2.2                   tidyselect_1.1.2
##  [61] rlang_1.0.5                  later_1.3.0
##  [63] AnnotationDbi_1.59.1         munsell_0.5.0
##  [65] BiocVersion_3.16.0           tools_4.2.1
##  [67] cachem_1.0.6                 cli_3.4.0
##  [69] generics_0.1.3               RSQLite_2.2.16
##  [71] evaluate_0.16                stringr_1.4.1
##  [73] fastmap_1.1.0                yaml_2.3.5
```

```
##  [75] knitr_1.40                    bit64_4.0.5
##  [77] purrr_0.3.4                   randomForest_4.7-1.1
##  [79] AnnotationFilter_1.21.0       KEGGREST_1.37.3
##  [81] Rbowtie_1.37.0                mime_0.12
##  [83] xml2_1.3.3                    biomaRt_2.53.2
##  [85] BiocStyle_2.25.0              compiler_4.2.1
##  [87] rstudioapi_0.14               filelock_1.0.2
##  [89] curl_4.3.2                    png_0.1-7
##  [91] interactiveDisplayBase_1.35.0 tibble_3.1.8
##  [93] stringi_1.7.8                 basilisk.utils_1.9.3
##  [95] GenomicFeatures_1.49.6        lattice_0.20-45
##  [97] ProtGenerics_1.29.0           Matrix_1.4-1
##  [99] vctrs_0.4.1                   pillar_1.8.1
## [101] lifecycle_1.0.1               BiocManager_1.30.18
## [103] data.table_1.14.2             bitops_1.0-7
## [105] httpuv_1.6.5                  rtracklayer_1.57.0
## [107] GenomicRanges_1.49.1          R6_2.5.1
## [109] BiocIO_1.7.1                  latticeExtra_0.6-30
## [111] promises_1.2.0.1              gridExtra_2.3
## [113] IRanges_2.31.2                codetools_0.2-18
## [115] dichromat_2.0-0.1             assertthat_0.2.1
## [117] SummarizedExperiment_1.27.2   rjson_0.2.21
## [119] GenomicAlignments_1.33.1      Rsamtools_2.13.4
## [121] S4Vectors_0.35.3              GenomeInfoDbData_1.2.8
## [123] parallel_4.2.1                hms_1.1.2
## [125] rpart_4.1.16                  grid_4.2.1
## [127] basilisk_1.9.6                rmarkdown_2.16
## [129] MatrixGenerics_1.9.1          biovizBase_1.45.0
## [131] Biobase_2.57.1                shiny_1.7.2
## [133] base64enc_0.1-3               interp_1.1-3
## [135] restfulr_0.0.15
```
