# Supplementary material for: A comprehensive Bioconductor ecosystem for the design of CRISPR guide RNAs across nucleases and technologies
Source: Nat Commun. 2022 Nov 2;13:6568. doi: 10.1038/s41467-022-34320-7 (PMC9630310; doi:10.1038/s41467-022-34320-7)
Supplement: Supplementary file 5 — Supplementary Software [file 41467_2022_34320_MOESM5_ESM.zip › SupplementarySoftware/Tutorial2_Building_A_Genome_Index.pdf]

Building genome indices off-target alignment

Jean-Philippe Fortin, Luke Hoberecht

Introduction

This vignette demonstrates how to build genome indices for the purpose of performing on- and off-target alignment. In particular, we show how to build such indices for the short read aligners bowtie (Langmead et al. 2009), as used by the `Rbowtie` and `crisprBowtie` packages, and BWA-backtrack (Li and Durbin 2009), as used by the `Rbwa` and `crisprBwa` packages. Note that BWA is not available for Windows users.

Generating a genome index file is time consuming, but only needs to be done once for a given genome.

Installation

See the Installation tutorial to learn how to install the `crisprBowtie` and `crisprBwa` packages.

Building a bowtie index

In the following example, we build a bowtie index for the human genome using the hg38 build. First, users will need to donwload the FASTA file from the UCSC genome browser. Here's the link: https://hgdownload.soe.ucsc.edu/goldenPath/hg38/bigZips/hg38.fa.gz

Next, assuming the `hg38.fa.gz` is located in the current directory, we build the bowtie genome index using the function `bowtie_build` from the `Rbowtie` package (which is installed when `crisprBowtie` is installed):

```
library(Rbowtie)
fastaFile <- "./hg38.fa.gz"
bowtie_build(fastaFile,
             outdir="./",
             force=TRUE,
             prefix="hg38")
```

This should take a couple of hours to run, and the resulting bowtie index files will be located in the folder `./hg38` and can be used to run bowtie alignment. See the crisprBowtie package to learn how to perform a bowtie alignment within R.

Building a BWA index

Building a BWA index is similar to building a bowtie index. Assuming the `hg38.fa.gz` is located in the current directory, we build the BWA genome index using the function `bwa_build_index` from the `Rbwa` package (which is installed when `crisprBwa` is installed):

```
library(Rbwa)
fastaFile <- "./hg38.fa.gz"
bwa_build_index(fastaFile,
                index_prefix="hg38")
```

This should take a couple of hours to run, and the resulting BWA index files will be located in the folder `./hg38` and can be used to run BWA alignment. See the crisprBwa package to learn how to perform a BWA alignment within R.

Building a transcriptome index

For applications using RNA-targeting nucleases such as CasRx, off-target search is performed against against transcriptomes rather than genomes. Building a transcriptome index works similar, except that we first need to generate a FASTA file containing the transcriptome sequences. This is easily accomplished with the function `getMrnaSequences` from the `crisprDesign` package, assuming that a gene model is provided, as well as a `BSgenome` object containing the DNA sequences for the hg38 genome (`BSgenome.Hsapiens.UCSC.hg38`).

We first load the necessary packages

```
library(BSgenome.Hsapiens.UCSC.hg38)
library(crisprDesign)
```

The `crisprDesignData` package (see Installation) contains a gene model annotation for the hg38 genome, and can be loaded using the following:

```
library(crisprDesignData)
data("txdb_human", package="crisprDesignData")
```

See the Gene annotation tutorial to learn more about how to build such gene annotation objects.

We will now extract mRNA sequences for all available transcripts:

```
txids <- unique(txdb_human$exons$tx_id)
mrnasHuman <- getMrnaSequences(txids,
                               bsgenome=BSgenome.Hsapiens.UCSC.hg38,
                               txObject=txdb_human)
```

This should take less than an hour to run. Once completed, we will write the extracted mRNA sequences to disk using the FASTA format. This can be accomplished using the `writeXStringSet` function from the `Biostrings` package:

```
library(Biostrings)
writeXStringSet(mrnasHuman,
                file="ensembl_human_104.fasta",
                format="fasta")
```

Note that the `seqnames` of this FASTA file are Ensembl transcript IDs instead of chromosomes. Once the FASTA file has been generated, the process for constructing either a bowtie or BWA index file is the same as described in the above sections.

Reproducibility

```
sessionInfo()
```

```
## R version 4.2.1 (2022-06-23)
## Platform: x86_64-apple-darwin17.0 (64-bit)
## Running under: macOS Catalina 10.15.7
##
## Matrix products: default
## BLAS:   /Library/Frameworks/R.framework/Versions/4.2/Resources/lib/libRblas.0.dylib
## LAPACK: /Library/Frameworks/R.framework/Versions/4.2/Resources/lib/libRlapack.dylib
##
```

```
## locale:
## [1] en_US.UTF-8/en_US.UTF-8/en_US.UTF-8/C/en_US.UTF-8/en_US.UTF-8
##
## attached base packages:
## [1] stats4    stats     graphics  grDevices utils     datasets  methods
## [8] base
##
## other attached packages:
##  [1] BSgenome.Hsapiens.UCSC.hg38_1.4.4 BSgenome_1.65.2
##  [3] rtracklayer_1.57.0                Biostrings_2.65.2
##  [5] XVector_0.37.0                    GenomicRanges_1.49.1
##  [7] GenomeInfoDb_1.33.5               IRanges_2.31.2
##  [9] S4Vectors_0.35.1                  crisprDesignData_0.99.17
## [11] crisprDesign_0.99.133             crisprScore_1.1.14
## [13] crisprScoreData_1.1.3             ExperimentHub_2.5.0
## [15] AnnotationHub_3.5.0               BiocFileCache_2.5.0
## [17] dbplyr_2.2.1                      BiocGenerics_0.43.1
## [19] crisprBowtie_1.1.1                crisprBase_1.1.5
## [21] crisprVerse_0.99.8                rmarkdown_2.15.2
##
## loaded via a namespace (and not attached):
##  [1] rjson_0.2.21                  ellipsis_0.3.2
##  [3] Rbowtie_1.37.0                bit64_4.0.5
##  [5] lubridate_1.8.0               interactiveDisplayBase_1.35.0
##  [7] AnnotationDbi_1.59.1          fansi_1.0.3
##  [9] xml2_1.3.3                    codetools_0.2-18
## [11] cachem_1.0.6                  knitr_1.40
## [13] jsonlite_1.8.0                Rsamtools_2.13.4
## [15] png_0.1-7                     shiny_1.7.2
## [17] BiocManager_1.30.18           readr_2.1.2
## [19] compiler_4.2.1                httr_1.4.4
## [21] basilisk_1.9.2                assertthat_0.2.1
## [23] Matrix_1.4-1                  fastmap_1.1.0
## [25] cli_3.3.0                     later_1.3.0
## [27] htmltools_0.5.3               prettyunits_1.1.1
## [29] tools_4.2.1                   glue_1.6.2
## [31] GenomeInfoDbData_1.2.8        dplyr_1.0.9
## [33] rappdirs_0.3.3                tinytex_0.41
## [35] Rcpp_1.0.9                    Biobase_2.57.1
## [37] vctrs_0.4.1                   crisprBwa_1.1.3
## [39] xfun_0.32                     stringr_1.4.1
## [41] mime_0.12                     lifecycle_1.0.1
## [43] restfulr_0.0.15               XML_3.99-0.10
## [45] zlibbioc_1.43.0               basilisk.utils_1.9.1
## [47] vroom_1.5.7                   VariantAnnotation_1.43.3
## [49] hms_1.1.2                     promises_1.2.0.1
## [51] MatrixGenerics_1.9.1          parallel_4.2.1
## [53] SummarizedExperiment_1.27.1   RMariaDB_1.2.2
## [55] yaml_2.3.5                    curl_4.3.2
## [57] memoise_2.0.1                 reticulate_1.25
## [59] biomaRt_2.53.2                stringi_1.7.8
## [61] RSQLite_2.2.16                BiocVersion_3.16.0
## [63] highr_0.9                     BiocIO_1.7.1
## [65] randomForest_4.7-1.1          GenomicFeatures_1.49.6
```

```
## [67] filelock_1.0.2               BiocParallel_1.31.12
## [69] rlang_1.0.4                  pkgconfig_2.0.3
## [71] matrixStats_0.62.0           bitops_1.0-7
## [73] evaluate_0.16                lattice_0.20-45
## [75] purrr_0.3.4                  GenomicAlignments_1.33.1
## [77] bit_4.0.4                    tidyselect_1.1.2
## [79] magrittr_2.0.3               R6_2.5.1
## [81] generics_0.1.3               DelayedArray_0.23.1
## [83] DBI_1.1.3                    pillar_1.8.1
## [85] KEGGREST_1.37.3              RCurl_1.98-1.8
## [87] tibble_3.1.8                 dir.expiry_1.5.0
## [89] crayon_1.5.1                 utf8_1.2.2
## [91] tzdb_0.3.0                   progress_1.2.2
## [93] grid_4.2.1                   blob_1.2.3
## [95] digest_0.6.29                xtable_1.8-4
## [97] httpuv_1.6.5                 Rbwa_1.1.0
```

References

Langmead, Ben, Cole Trapnell, Mihai Pop, and Steven L. Salzberg. 2009. "Ultrafast and Memory-Efficient Alignment of Short DNA Sequences to the Human Genome." *Genome Biology* 10 (3): R25. https://doi.org/10.1186/gb-2009-10-3-r25.

Li, Heng, and Richard Durbin. 2009. "Fast and Accurate Short Read Alignment with Burrows–Wheeler Transform." *Bioinformatics* 25 (14): 1754–60.
